# Calcitonin Gene-Related Peptide (CGRP) and Pituitary Adenylate Cyclase-Activating Polypeptide (PACAP) in Migraine Pathogenesis

**DOI:** 10.3390/ph15101189

**Published:** 2022-09-27

**Authors:** Casper Emil Christensen, Messoud Ashina, Faisal Mohammad Amin

**Affiliations:** 1 Danish Headache Center, Department of Neurology, Rigshospitalet Glostrup, Glostrup, 2600 Copenhagen, Denmark; 2 Department of Neurorehabilitation/Traumatic Brain Injury, Rigshospitalet, 2100 Copenhagen, Denmark

**Keywords:** migraine, neurovascular, headache, meningeal artery, cerebral artery, MRI, MR-angiography

## Abstract

Migraine is a prevalent and debilitating neurologic disorder. Advancements in understanding the underlying pathophysiological mechanisms are spearheading the effort to introduce disease-specific treatment options. In recent years this effort has largely focused on alteration of endogenous neuropeptide signaling, namely the peptides calcitonin gene-related peptide (CGRP) and pituitary adenylate cyclase-activating polypeptide (PACAP). Human studies into the pathophysiological underpinnings of CGRP and PACAP in migraine are manifold and here we review the works investigating these neuropeptides in patients suffering from migraine in order to elucidate the background for developing new treatment options for this vastly disabling disorder.

## 1. Introduction

Migraine is a multifaceted neurovascular disease and the second most prevalent neurologic disorder [1]. The diagnosis of migraine is based on a specific pattern of clinical features that includes both the headache and the various accompanying symptoms. These equally debilitating symptoms, present in varying degree during attacks, comprise nausea, vomiting, photophobia, and phonophobia [2]. A migraine attack is divided into phases starting with the prodromal (or the “premonitory”) phase where patients experience unusual tiredness, neck stiffness, and concentration difficulties, amongst other symptoms [3]. After the prodromal phase, which can last up to 48 h, around one in four individuals with migraine experience aura symptoms (transient neurological symptoms) which typically manifest as gradually progressing visual or hemisensory symptoms for up to 60 min preceding headache [2]. The third phase of migraine is the headache phase running 4 to 72 h, if left untreated, before finally entering the postdromal, or resolution phase [4]. Although not fully elucidated, the pathophysiological basis of migraine is believed to involve the trigeminal nerve and its connections to the cranial vasculature coining the term *trigeminovascular system* [5]. Release of neuropeptides upon activation of the trigeminovascular system may play a key role in migraine pathogenesis [6]. Whilst a plethora of regulatory molecules may be at play, calcitonin gene-related peptide (CGRP) and pituitary adenylate cyclase-activating polypeptide (PACAP) are both hypothesized to play important roles in migraine. Both CGRP and PACAP are powerful vasodilators and are both widely distributed in the trigeminovascular system [6,7]. Here we review clinical studies on the role of these neuropeptides in migraine pathophysiology.

## 2. Human Models of Migraine

A fundamental trait of migraine is the ability of certain endogenous and exogenous substances or conditions to trigger attacks. Patients with migraine report various triggers, e.g., certain foods and beverages, weather changes, sleep disturbances, and menstrual cycle [8]. This ability has been implemented in migraine research, where pharmacological triggers are used to provoke migraine attacks in a clinical setting to study all aspects of genesis, the course of an attack and pathophysiological properties [9]. In essence, a patient with migraine is administered a triggering agent, and attack incidence and properties are recorded in the following hours. Glyceryl trinitrate was the first substance proved to initiate migraine attacks mimicking the patient’s familiar attacks in a pioneering study almost 30 years ago [10]. In the following decades, a multitude of models have been introduced, among them are the models utilizing CGRP and PACAP to initiate migraine attacks.

## 3. Calcitonin Gene-Related Peptide

### 3.1. Triggering Headache and Migraine Attacks with CGRP

CGRP is an endogenous neuropeptide abundant in the trigeminovascular system [5]. Plasma levels of CGRP were first found to be elevated during migraine attacks compared to historical data from healthy controls [11]. These findings, however, were not reproduced in a paired setup where CGRP-levels were measured in the same patients during and between attacks [12]. When administered intravenously, infusion of CGRP induces migraine attacks in individuals with migraine [13]. These findings were reproduced in multiple provocation studies [14,15,16]. In healthy volunteers, CGRP infusion causes only a mild headache, which is usually not pulsating or accompanied by nausea or increased sensitivity to light and sound [17,18,19]. In migraine without aura, the first double-blinded placebo-controlled study reported that 33% of the patients developed delayed migraine attacks after CGRP (2.0 µg/min) compared to none after placebo [13]. The relatively low migraine induction rate with CGRP in the first study has later been attributed to the very strict attack criteria that were applied [20]. Since then, apt criteria for experimentally induced migraine attacks have been defined based on two categories: (1) headache which, apart from the 4 h minimum duration, is clinically indistinguishable from the internationally defined criteria, or (2) headache that mimics a patient’s usual attack and is successfully treated by abortive medication [7]. All CGRP provocation studies since the first one found higher induction rates, although using a lower dose of CGRP (1.5 µg/min) [14,15,16]. Calcitonin gene-related peptide elicits its effects by a second messenger, cyclic adenosine monophosphate (cAMP) and subsequent increase of intracellular Ca-ions via phosphorylation of protein kinase A (PKA) [21]. Sildenafil, a phosphodiesterase-5 inhibitor, also triggers migraine but via increase in cyclic GMP instead [22]. In a double-blinded cross-over study, comparing CGRP and sildenafil, 67% of patients suffered attacks after CGRP and 63% had attacks both after CGRP and after sildenafil [23]. This suggests, that a common cellular determinator downstream from the cyclic nucleotides, be they cAMP or cGMP, is paramount to the cellular mechanisms in migraine induction. 

**Figure 1:**
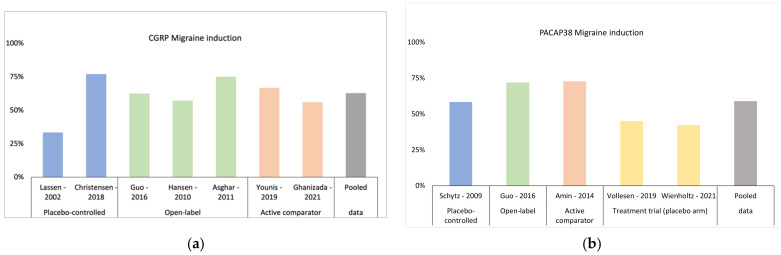
Migraine induction rates in the various provocation studies investigating migraine using calcitonin gene-related peptide (CGRP) (**a**), and pituitary adenylate cyclase-activating polypeptide-38 (PACAP38) (**b**), divided into study setups. “Active comparator” denotes the fact that each peptide was tested against another provocation model and “treatment trial” indicates that data has been extrapolated from the placebo arm of a study that sought to inhibit migraine induction by PA-CAP38.

Migraine provocation studies using CGRP have also emphasized the heterogeneity of the disease (Figure 1). Open-label CGRP trigger trials reported induction rates up to 75% [14,15]. These higher rates in open-label studies may reflect a nocebo effect in participants, emphasizing the strength in placebo-controlled trials in this type of study when induction rates are investigated [24]. Moreover, genetic make-up might also, in part, explain the difference in induction rates, as 75% of patients with higher genetic load (i.e., at least 14 migraine risk alleles) experienced attacks, compared to 52% in the group with low genetic load in yet another study [15]. Only one study investigated the effect of open-label CGRP in patients suffering from exclusively typical migraine with aura. Four of 14 patients (28%) developed aura symptoms, while 57% experienced a migraine attack without aura [16]. A similar open-label study in patients with familial hemiplegic migraine reported that CGRP induced neither aura symptoms nor migraine-like attacks [25]. Thus, available data suggest that the common subtypes of migraine (i.e., with and without aura) may not share underlying CGRP-related mechanisms with hemiplegic migraine.

### 3.2. CGRP’s Mode of Action

The role of CGRP in migraine pathophysiology has been extensively investigated during the past three decades [6]. In vascular smooth muscle cells, the CGRP-induced increase in intracellular Ca ions leads to relaxation and concomitant arterial dilation [26]. Interestingly, CGRP is involved in non-headache pain conditions [27], but intravenous infusion of CGRP does not cause non-cephalic pain in migraine patients or healthy volunteers without known headache or pain disorders [17,18]. The vasodilator effect of CGRP is, however, not limited to the intracerebral vasculature [28]. It is also unlikely that vasodilation *per se* may cause migraine attacks, as marked short-lasting vasodilation from vasoactive intestinal peptide infusion is not enough to induce migraine attacks [29]. Interestingly, both spontaneous and CGRP-induced unilateral migraine attacks were associated with ipsilateral cranial vasodilation [14,30]. Consequently, cranial artery dilation has been suspected of being a sign of activation of the trigeminal nociceptive afferents during migraine attacks [31]. If CGRP directly activates the trigeminal nociceptors or via a cascade of reactions remains to be investigated in vivo.

### 3.3. CGRP’s Site of Action

The CGRP receptor is a heterodimer complex consisting of the calcitonin receptor-like receptor and receptor activity-modifying protein 1 [32]. Full functional CGRP receptors are found in the human cerebral and middle meningeal (MMA) arteries [33], even though the human cerebral arteries were approximately 10 times more sensitive to CGRP compared to the MMA in vitro [33]. In preclinical studies the maximum response was more than 10 times higher after abluminal compared to luminal application of CGRP on the rat middle cerebral artery (MCA). The difference was ascribed to the blood-brain barrier (BBB) being an obstacle to the passage of CGRP from the blood to vascular smooth muscle cells [34]. The effect of CGRP on rat and human arteries is comparable [34]. Interestingly, high-resolution magnetic resonance angiography (MRA) revealed 10 times larger dilation of the MMA than the MCA after intravenous CGRP infusion in healthy volunteers [17]. A later and larger study reported an even more pronounced difference between the MMA (15.7%) and MCA (1.1%) at 30 min after CGRP infusion in healthy volunteers [18]. The findings indicate that the migraine-inducing effect of CGRP may be outside of the BBB. In support, neither of the antimigraine CGRP antagonist (olcegepant) nor the CGRP neutralizing antibody ALD405 were able to cross the BBB in a mouse model of migraine [35]. A PET imaging tracer targeting the CGRP receptor was used to show that the CGRP receptor antagonist, telcagepant, did not engage central CGRP receptor targets in rhesus monkeys in clinical dosing, further suggesting that the effect on migraine is driven by peripheral actions [36,37]. Taken together, CGRP seems to exert its migraine-inducing effects outside of the BBB and the anti-migraine effects of CGRP-targeted therapy might also be situated peripherally.

### 3.4. CGRP-Targeted Treatment

The above-mentioned implications of CGRP in migraine pathophysiology led to efforts in CGRP-targeted treatment options. Already, in the 1980s, it was shown that the 30-aminoacid CGRP fragment CGRP_8-37_ is a potent CGRP antagonist [38]. However, the peptide nature of this antagonist limited its potential for clinical use. A decade later, Boehringer patented a group of non-peptide CGRP antagonists, prompting the development of anti-migraine therapies [39]. A small molecule, the CGRP receptor antagonist (a so-called gepant) was the first anti-CGRP treatment to show promise in migraine therapy. Olcegepant showed efficacy in acute abortive treatment of migraine [40]. However, development was halted due to failure to produce an oral formulation. An oral small molecule CGRP antagonist, telcagepant, also met efficacy end points in clinical trials but development was stopped due to a small number of patients experiencing hepatotoxicity [41,42]. Four compounds are currently in production in the gepant family: rimegepant, atogepant, zavegepant, and ubrogepant. While initially developed and tested for the acute treatment of migraine attacks, several of the current gepants are also being tested preventively for migraine. Atogepant was approved in September 2021 for use in migraine prevention by the US Food and Drug Association (FDA). Daily administration of atogepant provides a therapeutic gain of 1.2, 1.4, and 1.7 monthly migraine days, respectively, with increasing doses [43]. Ubrogepant, also approved by the FDA, is used as abortive medication. One study found therapeutic gains in 2 h pain freedom of 7.4% and 9.4% using 50 or 100 mg doses of ubrogepant, respectively. Another measure of efficacy which is arguably a more patient-centered approach, is 2 h freedom from the most bothersome symptom. With respect to the most bothersome symptom, ubrogepant showed therapeutic gains of 10.8% and 9.9% with 50 mg and 100 mg doses, respectively [44]. Rimegepant is approved by the FDA for acute and preventive treatment of migraine. Rimegepant lowered monthly migraine days by 4.3 days compared to 3.5 days after placebo in a prevention setting, with administration of 75 mg tablets every other day for 12 weeks [45]. For acute treatment of migraine, 75 mg rimegepant resulted in 2 h post-administration pain freedom in 21% vs. 11% after placebo. The same study also evaluated 2 h freedom from the most bothersome symptom in 35% vs. 27%, after placebo [46]. Zavegepant is the only one of the four which is administered as a nasal spray for the acute treatment of migraine. While a 2019 press release announced zavegepant as efficacious in the acute treatment of migraine, both regarding 2 h pain freedom and 2 h freedom from the most bothersome symptom, the results from the study (NCT03872453) have not yet been published at the time of writing this review.

Parallel to the development of small molecule CGRP receptor antagonists, four different CGRP-targeted monoclonal antibodies (mAbs) have been developed for the treatment of migraine, namely erenumab, fremanezumab, galcanezumab, and eptinezumab. Erenumab binds specifically to the CGRP receptor, while the other three bind to the peptide. All four have been approved for use by both the FDA and the European Medicines Agency (EMA). Multiple randomized clinical trials have evaluated the safety and efficacy of all four anti-CGRP mAbs with some differences in patient cohorts, as well as effect parameters. Selected phase 2 and 3 studies are reviewed below. Mean decline in monthly migraine days range from around 2 to 8 days (for extensive review, see Charles and Pozo-Rosich, The Lancet, 2019 [47] and Dodick D, Cephalalgia, 2019 [48]).

#### 3.4.1. Erenumab

Erenumab is the first mAb that binds to the CGRP receptor and was approved by the FDA in 2018 [49]. The clinical application of erenumab (commercially labelled Aimovig), is for the preventive treatment of episodic and chronic migraine, administered in monthly subcutaneous injections. The efficacy of erenumab has been shown in various phase 2 and 3 studies. Erenumab in three different dosages (7 mg, 21 mg, and 70 mg) was compared to placebo in a randomized, multicenter, placebo-controlled phase 2 study that included 483 adults with migraine [50]. Erenumab 70 mg decreased the number of monthly migraine days (MMD) by 3.4 days compared to 2.3 days with placebo [50]. Two phase 3 trials confirmed the positive effect of erenumab using both 70 mg and 140 mg dosages. The ARISE trial, which enrolled patients with episodic migraine, compared 70 mg erenumab to placebo in the third month of a three-month treatment phase and found a change in MMD of −2.9 days with erenumab and −1.8 days in the placebo group [51]. The STRIVE trial randomized patients to receive monthly doses of erenumab 70 mg, 140 mg, or placebo with a change in MMD of −3.2 days (70 mg group), −3.7 days (140 mg group), and −1.8 days (placebo group) [52].

#### 3.4.2. Fremanezumab

Fremanezumab is a humanized antibody that selectively binds to the CGRP ligand. Commercially labelled Ajovy, fremanezumab is also administered as monthly subcutaneous injections [48]. Two randomized placebo-controlled phase 3 studies evaluated the efficacy of fremanezumab in patients with episodic and chronic migraine [53,54]. In patients with episodic migraine, 875 participants were randomized 1:1:1 to monthly injections (baseline, week 4, and week 8) of fremanezumab 225 mg, or a starting dose of fremanezumab 675 mg followed by monthly placebo injections at weeks 4 and 8, or monthly placebo injections at baseline and weeks 4 and 8. From baseline to week 12 the least squares mean change in MMD was −3.7 days for monthly dosing, −3.4 days for initial high dose, and −2.2 days for placebo [53]. In a trial of 1130 chronic migraine patients, participants were randomized 1:1:1 to monthly dosing of fremanezumab (675 mg at baseline and 225 mg at weeks 4 and 8), or a single high-dose fremanezumab (675 mg) followed by placebo at weeks 4 and 8, or matching placebo. The primary end point was change in headache days (not migraine days) and the treatment regimens yielded a change from baseline to week 12 of −4.6 days for monthly dosing of fremanezumab, −4.3 days for the initial high dose, and −2.5 days for placebo [54].

#### 3.4.3. Galcanezumab

Galcanezumab (labelled Emgality), is a mAb targeting the CGRP ligand, also administered as monthly subcutaneous injections. EVOLVE-1 and EVOLVE-2 were phase 3 studies evaluating galcanezumab versus placebo in patients with episodic migraine. EVOLVE-1 randomized 862 patients 1:1:2 to galcanezumab 120 mg monthly, 240 mg monthly, or placebo. The 120 mg group had a reduction in MMD of 4.7 days, the 240 mg group had a 4.6-day reduction, and placebo a 2.8-day reduction [55]. EVOLVE-2 compared similar dosing regimens as EVOLVE-1 to placebo and found reductions in MMD of 4.3, 4.2, and 2.3 days using 120 mg, 140 mg, and placebo administrations, respectively [56]. In patients with chronic migraine, the phase 3 REGAIN trial found monthly injections with 120 mg galcanezumab (with a 240 mg loading dose) to reduce MMD by 4.8 days, and 240 mg dosing reduced MMD by 4.6 days, compared to 2.7 days with placebo [57].

#### 3.4.4. Eptinezumab

Eptinezumab (commercially labelled Vyepti) is the only anti-CGRP mAb available in an intravenous (i.v.) formulation for the prevention of migraine [48]. Two phase 3 trials evaluated i.v. eptinezumab in episodic (PROMISE 1) and chronic (PROMISE 2) migraine [58,59]. PROMISE 1 evaluated eptinezumab vs. placebo in three different dosages: 30 mg, 100 mg, and 300 mg as i.v. formulation every 12 weeks. The 100 mg and 300 mg dosages met the primary end point with change in MMD from baseline to week 12 by −3.9 days in the 100 mg group, −4.3 days in the 300 mg group, and −3.2 days after placebo [58]. Patients with chronic migraine were included in PROMISE 2, which found change in MMD of −5.6 days after placebo, −7.7 days in the 100 mg eptinezumab group, and −8.2 days in the 300 mg group [59].

### 3.5. Clinical Relevance of Anti-CGRP Antibody Therapy

The collective data on safety and efficacy of anti-CGRP antibody treatments for migraine prevention have brought them from bench to bedside and into the headache clinics as new tools for clinicians treating migraine. This is evident also, in a recent update from the European Headache Federation, which provides a guideline for the use of anti-CGRP antibody treatment in migraine [60].

## 4. Pituitary Adenylate Cyclase-Activating Polypeptide

### 4.1. Triggering Headache and Migraine Attacks with PACAP

The pituitary adenylate cyclase-activating polypeptide-38 (PACAP38) and pituitary adenylate cyclase-activating polypeptide-27 (PACAP27) are found all over the human body [61]. These peptides are structurally and functionally related and they act via three transmembrane G-protein-coupled receptors, namely the VPAC_1_, VPAC_2_ and the PAC_1_ receptors [61]. Intravenous infusion of PACA38 and PACAP27 in healthy volunteers cause only a slight headache or rather a feeling of pressure in the head immediately after the start of infusion. In a small group of patients this feeling can last a few hours [62,63,64]. Using identical experimental settings, but in migraine without aura patients instead of healthy volunteers, intravenous infusion of both peptides can cause migraine attacks indistinguishable from the patients’ naturally triggered attacks [29,62,65]. Double-blinded placebo-controlled studies reported migraine attacks in 58% after PACAP38 [62] and in 55% after PACAP27 [65]. The migraine attack induction rate was even higher in open-label trial in patients with and without familial aggregation of migraine. Here, 75% of the patients with at least two first-degree relatives with migraine developed attacks, while 70% of the patients with less than two first-degree relatives with migraine experienced a migraine attack. There was no statistical difference between the two groups [66]. In another double-blind crossover study comparing PACAP38 with vasoactive intestinal peptide (VIP), the attack induction was 75% after PACAP38 and 18% after VIP [29]. Interestingly, in a study where patients received PACAP38 followed by either sumatriptan, a 5-HT_1B/1D_-receptor agonist, or placebo (i.e., isotonic saline), the attack induction rate was 42% on the placebo day [67] (Figure 1). The differences in the induction rates may be explained by the nocebo response, which is assumed to be 15.5% in human migraine provocation studies [24].

### 4.2. PACAP’s Mode of Action

It has been discussed how PACAP exerts its migraine inducing effect. PACAP and its receptors are widely found in the human body, including the brain as well as smooth muscle cells in the arteries [61,68]. Two main mechanisms of PACAP-induced migraine attacks have been proposed, including long-lasting vasodilation and mast cell degranulation [29]. PACAP and VIP share their receptors, but PACAP38 binds to the PAC_1_ receptor with much higher affinity than does VIP [61]. Intravenous infusion over 20 min of both PACAP38 and VIP in equivalent doses caused equal responses in the middle meningeal artery (MMA) and the middle cerebral artery (MCA) during the infusion phase, but PACAP38-induced dilation of the MMA was longer lasting. The rate of migraine attack induction was also significantly higher after PACAP38 than after VIP [29]. Subsequently, a long-lasting infusion of VIP over 120 min caused attack induction in 71% of the patients [69]. In addition, early blockade of PACAP38-induced dilation of the superficial temporal artery (STA) with sumatriptan was accompanied by significantly reduced attack rate, at 15%, compared to 42% after placebo [67]. The effect of sumatriptan is mainly vasoconstriction [70] rather than counteracting histamine secretion from mast cells. Clemastine, a H_1_-antihistamine, had no effect on PACAP38-induced migraine attacks in a placebo-controlled study [71]. Moreover, in healthy volunteers, pre-treatment with ketorolac, a non-steroid anti-inflammatory drug, and sumatriptan 20 min before infusion of PACAP38 resulted in headache in 94% in the group treated with ketorolac and in 76% in the group treated with sumatriptan. In the same study, both ketorolac and sumatriptan were also administered 90 min after PACAP38 infusion. Here, 94% developed headache in the ketorolac group and 100% in the sumatriptan group [72]. As headache in healthy volunteers and migraine in migraine patients are phenotypically, and most likely pathophysiologically different, studies in migraine patients are needed to translate these findings with ketorolac into migraine pathophysiology. The VPAC_1_ and VPAC_2_ receptors seem to be responsible for vasodilation [73,74], while the PAC_1_ receptor seems to be involved in mast cell degranulation [75]. Moreover, the monoclonal antibody AMG 301, targeting the PAC_1_ receptor, recently failed to prevent migraine [76]. Collectively, the mode of action by which PACAP38 induces migraine attacks in patients seems to be associated with the vascular effects of PACAP38 rather than inflammation.

### 4.3. PACAP’s Site of Action

A common trait of all pharmacological migraine triggers, including the PACAPs, is that they are all potent vasodilators [6,7,69]. This justifies the focus on vasodilation when investigating underlying mechanisms for PACAP-induced migraine attacks. PACAP receptors are found in meningeal [77] as well as cerebral [68] arteries. In vitro studies investigating rat middle cerebral arteries reported relaxation after abluminal, but not luminal application of PACAP38. The difference was ascribed integrity of the blood-brain barrier (BBB). In the same study, authors reported no difference between rat and human cerebral arteries regarding dilation after abluminal application of PACAP38 [34]. In the normal functioning BBB, both PACAP38 and PACAP27 are able enter the endothelial cells but are then either rapidly degraded or they efflux back from the blood again [78]. Human studies reported intact BBB in migraine with and without aura [79,80,81]. Based on these, the effect of PACAPs on the meningeal and cerebral arteries may represent peripheral and central sites of action of PACAP. In human studies, using high-resolution magnetic resonance angiography (MRA), vascular changes have been thoroughly investigated before and after infusion of both PACAP27 and PACAP38 in healthy volunteers and migraine patients. In healthy volunteers, PACAP27 induced dilation of the MMA by some 26% (extrapolated from figure), while the MCA circumference changes by −2.5% (extrapolated from figure) 20 min after start of infusion [64]. In a similar study with PACAP38, the same pattern was reported with 23.4% MMA dilation and 1.5% MCA dilation [63]. In migraine without aura patients, the MMA dilated 27.3%, whereas the MCA change was −0.4% after 20 min of PACAP38 infusion [29]. There is a perfect consistency across all MRA studies of the vascular effect of PACAP. The MRA method used with the given numbers of participants was able to detect at least 12% dilation of both measured arteries [82]. Overall, the available evidence strongly suggests that PACAP’s site of action is outside of the BBB and most probably associated with changes of the meningeal arteries.

## 5. Conclusions

Both CGRP and PACAP play integral roles in the pathophysiology of migraine. While a tremendous amount of work has already gone into the study of these peptides, from their first discovery to the introduction of CGRP-targeted therapy in headache clinics, investigations into their pathomechanisms are still on-going. Each step towards a better understanding of neuropeptide function in migraine is a step towards treatment optimization for the one billion sufferers worldwide, and a search through clinicaltrials.gov conducted in April 2022 revealed that >40 studies are currently in the pipeline to improve our knowledge.

## Data Availability

Data sharing not applicable.
